# Comprehensive genomic profiling requires a blended ecosystem of learning healthcare and clinical trials

**DOI:** 10.1038/s41416-025-03009-1

**Published:** 2025-04-18

**Authors:** Albrecht Stenzinger, C. Benedikt Westphalen, Jan Budczies, Daniel Kazdal, Carolin Ploeger, Christian Altbürger, Matthias Evert, Nisar Malek, Peter Schirmacher, Frederick Klauschen

**Affiliations:** 1 Center for Personalized Medicine (ZPM), Heidelberg, Germany; 2 Institute of Pathology, Heidelberg University Hospital, Heidelberg, Germany; 3 Comprehensive Cancer Center and Department of Medicine III, Ludwig Maximilian University of Munich, Munich, Germany; 4 Institute of Pathology, University of Regensburg, Regensburg, Germany; 5 Center for Personalized Medicine (ZPM), Tübingen, Germany; 6 Department for Internal Medicine 1, Tübingen University Hospital, Tübingen, Germany; 7 Institute of Pathology, Ludwig Maximilian University of Munich, Munich, Germany; 8 BIFOLD-Berlin Institute for the Foundations of Learning and Data, Berlin, Germany

**Keywords:** Cancer genomics, Diagnostic markers, Targeted therapies

With great interest we read the excellent paper by Leung et al. [1] reporting on the use of whole genome sequencing (WGS) for cancer patients within the 100,000 Genomes Project.

Leung et al. showed that out of 4830 patients, 3067 could be sequenced and 377 received therapeutic recommendations, which resulted in a different clinical management for 17 patients. The fact that clinical management changed for less than 1% of patients for whom WGS diagnostics was performed is noteworthy. While follow-up data are not presented, the overall direct therapeutic impact in the cohort can be considered minimal. In addition, WGS analysis and discussion in the interdisciplinary board led to 141 recommendations regarding germline variants including *DPYD* variants implicated in 5-FU metabolism. Unfortunately, data on the actual clinical implications and potential changes in patient management are missing.

Apart from exploring the clinical utility of WGS [2], this thoroughly collected real-world data set is informative as it details the current bottlenecks in the implementation of comprehensive molecular diagnostics to inform therapeutic management in a publicly funded health care system—a strategy that is currently being pursued by many national networks [3]. The results presented by Leung and colleagues also show that some of the challenges encountered at the beginning of the study have improved over time, for example the quality control failure rate decreased and the initial median turnaround time of 16 weeks was reduced to 4 weeks. While one can only speculate about the specific reasons that led to these individual improvements, changes in organisational and technical processes, the establishment of network structures and the prevention of communication silos presumably played a decisive role.

**Fig. 1:**
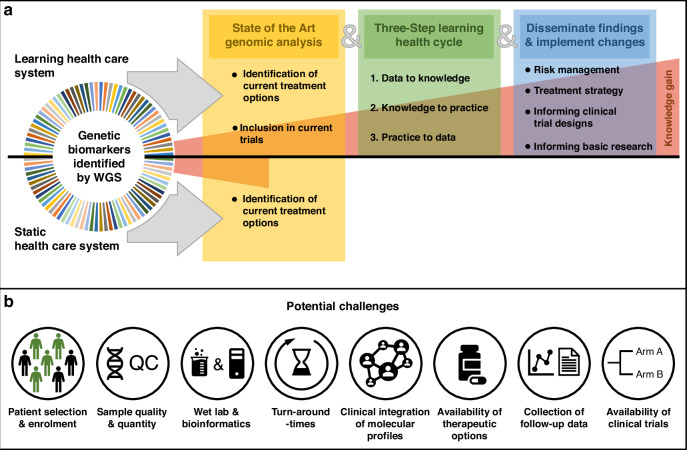
A learning healthcare system for comprehensive molecular profiling. **a** Implementation of genomic profiling for cancer patients requires a learning healthcare approach and integration with clinical trials for evidence generation, **b** Common challenges range from technical considerations to the availability of clinical trials.

Major challenges that can be distilled from the study include i) selection and enrolment criteria, ii) the type, quantity and quality of tumour tissue samples and extracted nucleic acids, iii) the type and extent of wet-lab and bioinformatic analyses, iv) turn-around-times of diagnostics including sample logistics, v) a well-trained healthcare workforce integrating the results of molecular profiling into clinical management vi) availability of therapeutic options as well as assigning evidence level in off-label scenarios, vii) collection of standardised follow-up data outside clinical trials, viii) availability of clinical trials (Fig. 1).

Considering several published cost calculation models [4–6], sequencing costs for 3067 patients alone could amount to 10 million euros or more, indirectly mandating the need for a balanced cost-benefit analysis. This scenario not only points to the intricate interplay between costs and reimbursement, which significantly influences the use of molecular profiling in a health care system, but may also facilitate a discussion around potential opportunity costs from an economic perspective.

While WGS offers a very comprehensive approach potentially covering all current drug targets and biomarkers identifiable by sequencing and particularly enables the read-out of complex biomarkers and copy number profiles at high accuracy, the availability of fresh tissue, and low tumour cellularity may also impair diagnostic workflows and limit results. Another important aspect that translates directly to the validity of the analysis is the size of the genomic region covered by WGS, which is ~100 and ~3000 times larger compared to whole exome sequencing (WES) and comprehensive panel sequencing, respectively. To reduce costs, lower sequencing depth (e.g. 80–100×) is generally used for WGS profiling of solid tumours, although comparable depths to panel sequencing (e.g. 250–600x) would be technically possible. Low read depth reduces the sensitivity of any sequencing approach, as variants with a low variant allele fraction in a given sample may not be validly detected. This may affect subclonal genetic alterations and any biomarker that includes those (e.g. tumour mutational burden (TMB), microsatellite instability (MSI), homologous recombination deficiency (HRD), resistance mutations), but may even affect clonal (truncal) driver mutations in samples with low tumour cell content. Further technological developments, which allows the use of formalin-fixed paraffin-embedded material, as well as significantly lowering overall costs would support sufficiently deep and broad sequencing in samples with e.g. low tumour cell content, are important in this regard. WGS also necessitates comprehensive data analysis and interpretation. The content of virtual panels (gene lists) that focus on therapeutic targets, is a crucial consideration as it limits analysis burden while meeting clinical requirements.

It should also be noted that the detection of those genetic aberrations that led to therapeutic recommendations in the study did not necessarily require WGS. Leung et al. investigated variants in 143 actionable genes of solid and in 118 actionable genes of haematological malignancies resulting in treatment recommendations based on alterations in 45 genes. These genes could be covered by a comprehensive gene panel (for example, 85%, 85%, and 96% of the three gene sets are covered by the TruSight Oncology (TSO) 500 DNA panel). The germline variants in susceptibility genes reported by Leung et al. could have also been identified by a comprehensive panel sequencing approach but WGS certainly has the advantage of uncovering more complex structural germline variants involving intronic and intergenic regions which may be missed by panel sequencing alone. Immune checkpoint inhibition because of high TMB was the most common recommendation among licensed and unlicenced treatments. While TMB measurement is feasible using comprehensive panel sequencing, panel TMB estimates are afflicted by a stochastic error that can lead to the false classification of tumours close to the cutpoint of 10 mut/Mbp resulting in inferior predictive performance compared to WES or WGS [7]. In conclusion, while the overwhelming majority of clinical trials that led to approval of drug-biomarker matches - including checkpoint blockade—TMB [8]—are based on panel-based next-generation sequencing (NGS), potential advantages of WGS include the following three main aspects: i) panel redesign can be avoided when new addressable genes emerge, ii) structural genomic variations can be comprehensively revealed, and iii) complex biomarkers can be measured more accurately.

The decisive factor for the success of a sequencing-guided precision medicine approach is the interpretation of molecular changes with regard to their clinical actionability. Classification schemes such as the ESMO Scale for Clinical Actionability of molecular Targets (ESCAT) [9, 10] allow to capture the level of evidence for alteration-drug matches, help to reveal additional treatment options, and facilitate standardised communication among stakeholders.

This being said, comprehensive standardised data sets obtained by WGS can be of substantial value in a learning health care environment. Supplementing classical clinical trials, this real-world approach systematically combining clinical care and research integrates sequencing and clinical outcome data to identify molecularly defined groups of patients associated with different clinical outcomes. Results derived from such analyses may lead to hypotheses generation for basic research and fuel the development of specific clinical trials aiming at evidencing certain observations. Similarly, comprehensive molecular profiling, when coupled with a comprehensive clinical trial infrastructure, can support trial enrolment in a one-size fits all approach without employing various assays requiring expensive and time-consuming validations. Lastly, comprehensive sequencing data may also provide information beyond therapy prediction supporting cancer typing as well as genetic counselling.

To conclude, studies like the one conducted by Leung et al. [1] are paramount because they generate data that are critically required to meaningfully position and implement comprehensive genomic profiling, such as WGS, in routine clinical care. However, the study’s results also underline the importance of embedding comprehensive diagnostic profiling into a permissive healthcare ecosystem that is based on the concept of learning health care and integrated healthcare solutions including the development of clinical trials (Fig. 1). Only then we will be able to measure clinical utility and leverage the full potential of such technologies.
